# Feasibility of Intravoxel Incoherent Motion (IVIM) and Dynamic Contrast-Enhanced Magnetic Resonance Imaging (DCE-MRI) in Differentiation of Benign Parotid Gland Tumors

**DOI:** 10.3390/biology11030399

**Published:** 2022-03-04

**Authors:** Karolina Markiet, Anna Glinska, Tomasz Nowicki, Edyta Szurowska, Boguslaw Mikaszewski

**Affiliations:** 1 2nd Department of Radiology, Medical University of Gdansk, 80-214 Gdansk, Poland; anna.glinska@gumed.edu.pl (A.G.); tomasz.nowicki@gumed.edu.pl (T.N.); edyta.szurowska@gumed.edu.pl (E.S.); 2 Department of Otolaryngology, Medical University of Gdansk, 80-214 Gdansk, Poland; boguslaw.mikaszewski@gumed.edu.pl

**Keywords:** IVIM, DCE-MRI, DWI, parotid gland tumors, pleomorphic adenoma, Warthin tumor

## Abstract

**Simple Summary:**

Multiparametric magnetic resonance imaging gains recognition in the diagnostic algorithm of salivary gland tumors, providing data from different diffusion-weighted imaging (DWI) models and dynamic contrast enhanced sequences (DCE-MRI). The aim of this prospective study is to identify quantitative intravoxel incoherent motion and DCE-MRI parameters of the most frequent benign parotid tumors, pleomorphic adenomas and Warthin tumors, to compare their utility and diagnostic accuracy. With a precise pre-treatment diagnostic tool, patients can potentially avoid unnecessary diagnostic procedures and be offered optimal treatment.

**Abstract:**

Aim: The aim of this prospective study is to identify quantitative intravoxel incoherent motion and dynamic contrast-enhanced magnetic resonance imaging parameters of the most frequent benign parotid tumors, compare their utility and diagnostic accuracy. Methods: The study group consisted of 52 patients with 64 histopathologically confirmed parotid focal lesions. Parametric maps representing apparent diffusion coefficient (ADC), pure diffusion coefficient (D), pseudo-diffusion coefficient (D*), perfusion fraction (FP) and transfer constant (Ktrans), reflux constant (Kep), extra-vascular extra-cellular volume fraction (Ve), and initial area under curve in 60 s (iAUC) have been obtained from multiparametric MRI. Results: Statistically significant (*p* < 0.001) inter-group differences were found between pleomorphic adenomas (PA) and Warthin tumors (WT) in all tested parameters but iAUC. Receiver operating characteristic curves were constructed to determine the optimal cut-off levels of the most significant parameters allowing differentiation between WT and PA. The Area Under the Curve (AUC) values and thresholds were for ADC: 0.931 and 1.05, D: 0.896 and 0.9, Kep: 0.964 and 1.1 and Ve: 0.939 and 0.299, respectively. Lesions presenting with a combination of ADC, D, and Ve values superior to the cut-off and Kep values inferior to the cut-off are classified as pleomorphic adenomas. Lesions presenting with combination of ADC, D, and Ve values inferior to the cut-off and Kep values superior to the cut-off are classified as Warthin tumors. Conclusions: DWI, IVIM and quantitative analysis of DCE-MRI derived parameters demonstrated distinctive features of PAs and WT and as such they seem feasible in differentiation of benign parotid gland tumors.

## 1. Introduction

Three to six percent of tumors within the head and neck arise in the salivary glands [1], the majority within the parotid glands. Up to 80% of tumors within the parotids are benign with the two most common types being Warthin tumors (WTs) and pleomorphic adenomas (PAs). Despite both being benign lesions, they present with different clinical course if left untreated. Malignant transformation is reported in 1.9–23.3% of untreated PAs, whereas it is less than 1% for WTs [1,2]. Thus, WTs can be surveilled if asymptomatic, while surgery is a method of treatment for pleomorphic adenomas. The aim of surgery is to obtain a complete resection with clear histopathological margins, as recurrence rate after enucleation in up to 50% [2,3]. Additionally, special attention should be given to lesions within the deep lobe as they are not only difficult to diagnose in cytology due to accessibility issues but also demand special caution during operation in order to preserve surrounding structures, especially facial nerves. Hence, a precise pre-treatment diagnosis is essential. 

Reported overall sensitivity of salivary gland fine needle aspiration cytology (FNAC), which usually serves as a first-line diagnostic procedure, ranges from 86% to 100%, with specificity from 90% to 100% [4]. Sensitivity and specificity of FNAC, however, depend on technical experience of both person performing the biopsy and the evaluating pathologist, quality of cytologic preparations, morphological heterogeneity of the lesions, presence of cystic components and lesion location (with limited access when the lesion is in the deep lobe) [4]. 

Multiparametric magnetic resonance imaging (mpMRI) gains recognition in the diagnostic algorithm of salivary gland tumors, providing data from different diffusion-weighted imaging (DWI) models and dynamic contrast enhanced sequences (DCE-MRI) [5,6,7,8,9]. Diffusion-weighted imaging reflects the cell density of the tissue. The most used quantitative parameter of DWI, apparent diffusion coefficient (ADC), relates to the microscopic diffusion of the water molecules and is assessed in a mono-exponential model; its role seems to be well established in radiological clinical practice [5]. The intravoxel incoherent motion (IVIM) model proposed by LeBihan separates the perfusion-related parameters (pseudo-diffusion coefficient D* and perfusion fraction FP) and the diffusion-related parameters (pure diffusion coefficient D) by employing a bi-exponential analysis [10,11,12]. Previous studies proved the utility of IVIM in head and neck tumors’ differentiation [13,14,15,16,17,18], evaluating lymph nodes [19,20,21,22], monitoring outcome and/or treatment response [23,24], in the assessment of radiation-induced changes [25], and evaluating inflammatory processes, such as Sjögren’s syndrome [26,27]. Additionally, the potential role of IVIM analysis in parotid gland oncology seems to be increasing [5]. Semi-quantitative analysis of the contrast enhancement pattern in DCE-MRI reflected as time intensity curves is widely employed in salivary gland tumor differentiation [5,28]. The utility of quantitative data derived from DCE-MRI such as Ktrans (transfer constant), Kep (reflux constant), Ve (extra-vascular extra-cellular volume fraction), and iAUC (initial area under curve in 60 s) is now under ongoing research [29,30,31,32,33]. 

The aim of this prospective study is to identify quantitative IVIM and DCE-MRI parameters of the most frequent benign parotid tumors and compare the utility and diagnostic accuracy of those parameters. We believe that assessment of well-established features such as ADC values and time intensity curves together with novel quantitative parameters derived from IVIM and DCE-MRI will reveal tumor biology and hence provide significant information on tumor characteristics. 

## 2. Materials and Methods

### 2.1. Study Group 

Eighty-eight consecutive patients referred to The Department of Otolaryngology with major salivary gland nodules revealed in physical examination and/or in ultrasound underwent parametric magnetic resonance examination of the parotid glands in our institution between July 2020 and October 2021. Inclusion criteria were: 18+ years of age, the presence of at least one focal lesion within parotid glands other than of inflammatory origin/cyst and the absence of contraindications to magnetic resonance imaging/intravenous contrast agent administration. FNAC performed less than two weeks prior to MRI was an exclusion criterium; in case of one patient biopsy was performed two days after mpMRI. Patients with a history of radiotherapy in the head and neck region were not enrolled. 

After the initial analysis, 31 patients were excluded from further evaluation due to the presence of inflammatory lesions (6 pts), the presence of cysts (2 pts), submandibular gland lesions (2 pts), deviation from the examination protocol (5 pts), and a lack of post-operative histopathology examination results (8 pts). In eight patients, mpMRI did not confirm the presence of a focal lesion. We have further excluded 5 patients with malignant lesions due to a small number of lesions as well as group heterogeneity (1 muco-epidermoid carcinoma, 1 adenoid cystic carcinoma, 1 acinic cell carcinoma, 1 lymph node metastasis, 1 diffuse large B-cell lymphoma).

Final study cohort consisted of fifty-two patients with 64 histopathologically confirmed focal lesions within parotid glands: 33 women (mean age 57.67, age range 21–67 years old) and 19 men (mean age 53.05, age range 24–80 years old)—Table 1. Nine patients had multiple lesions (from 2 to 3 foci). All the patients underwent surgery and histopathology examination results, which were treated as a reference standard, revealed 31 Warthin tumors, 27 pleomorphic adenomas, 3 myoepitheliomas and 3 basal cell adenomas (Table 1). 

### 2.2. Imaging Protocol

All examinations were acquired with a 1.5 T scanner (Siemens Magnetom Sola, Erlangen, Germany) with a multi-array head-coil according to the examination protocol detailed in Table 2. Diffusion-weighted sequence was performed with 13 different b-values (0, 10, 20, 30, 50, 80, 100, 300, 400, 500, 800, 1000, 2000 s/mm^2^). Dynamic contrast-enhanced sequence was obtained with 36 consecutive scans with a temporal resolution of 6 s after intravenous contrast agent (CA) injection (Gadobutrol; Gadovist, Bayer Schering Pharma, Leverkusen, Germany) at a dose of 0.1 mL/kg body weight and flow rate of 3 mL/s followed by a flush of 20 mL saline solution at the same rate. CA was administered at the start of the first post-contrast scan with the use of an automatic injection system. No adverse reactions were reported. 

Informed consent was obtained from all patients involved in the study. The study was conducted according to the guidelines of the Declaration of Helsinki and approved by the Institutional Independent Ethics Committee for Scientific Research (NKBBN/26/2021). 

### 2.3. Image Analysis

Images have been independently evaluated by two radiologists with over 5 years of experience in head and neck radiology (KM—observer 1, TN—observer 2) blinded to the final histopathological results. Tumors have been identified on axial T1-weighted Turbo Spin Echo images. Initial morphological evaluation was followed by assessment of ADC values and time intensity curves (TICs). In order to assess apparent diffusion coefficient values, regions of interest (ROIs) were drawn manually on ADC maps by each observer independently over the entire tumor at an axial cross-section where the tumor maximum diameter was previously measured, carefully avoiding cystic areas and post biopsy hemorrhage (in case of two patients). ROIs were then copied within dynamic contrast-enhanced images in order to construct TICs. With concordance to the literature [34] TIC patterns were categorized as follows: type A—progressive contrast enhancement with delayed time to peak enhancement (T_peak_ over 120 s) and a poor washout (less than 10% at 5 min); type B—early enhancement (T_peak_ prior to 120 s) and high washout pattern (WR over 30% at 5 min); and type C—early enhancement (T_peak_ prior to 120 s) and low washout pattern (WR of less than 30% at 5 min). A flat curve without enhancement has not been observed within our cohort. The range and average ROI size were as follows: observer 1: range 0.11–7.34 cm^2^, mean 1.7 cm^2^, observer 2: range 0.10–7.03 cm^2^, mean 1.65 cm^2^, respectively. 

#### 2.3.1. Intravoxel Incoherent Motion (IVIM) Analysis

IVIM analysis was performed with commercially available software *syngo.via* Frontier MR Body Diffusion Toolbox (Siemens, Erlangen, Germany) with an assumption of bi-exponential signal evolution model and according to implemented algorithm 3 for full non-linear fitting of extracted IVIM-related features. ROIs were manually set within the same areas as those used in previous ADC/TICs analysis into each of constructed IVIM maps. In addition, correlating ROIs were set in the contralateral normal parotid gland parenchyma for reference. The following parameters were assessed and further statistically analyzed: D (pure diffusion coefficient), D* (pseudo-diffusion coefficient) and FP (perfusion fraction). 

#### 2.3.2. Quantitative DCE-MRI Analysis

Analyses were performed with a vendor-provided software *syngo.via* Tissue 4D (Siemens, Erlangen, Germany). We based our analysis on the Tofts model generating pixel-based maps from TICs with constant T1 (assumed T1 of 1300 ms; value obtained in our study as well as supported by literature [34]). Arterial input function (AIF) was set to intermediate [35], providing with an optimal median chi2 value at processing 0.021 (range 0.006–0.11). For quantitative analysis, the same 2 observers independently copied and pasted ROIs to each of permeability maps, obtaining values of Ktrans (transfer constant), Kep (reflux constant), Ve (extra-vascular extra-cellular volume fraction), and iAUC (initial area under curve in 60 s). As previously, correlating ROIs were set in the contralateral parotid gland for reference.

#### 2.3.3. Statistical Analysis

With the use of SPSS Statistica software (IBM Corporation, Armonk, NY, USA) a non-linear (non-Gaussian) distribution of variables (ADC, D, D*, Fp, Ktrans, Kep, iAUC) was confirmed. Further non-parametric tests were used for analysis of the variables. Inter-observer agreement levels were measured with intra-class correlation coefficient (ICC). ICCs were regarded as follows: values less than 0.5 indicate poor reliability, values between 0.5 and 0.75—moderate reliability, values between 0.75 and 0.9—good reliability and values greater than 0.90 indicate excellent reliability [36]. With good and excellent ICCs median values and interquartile range were calculated for each variable. Kruskal–Wallis test was used for evaluation of independent samples relations between Warthin tumors, pleomorphic adenomas and healthy parotids. Significance level was obtained at 0.05. Values of significance for multiple tests was corrected by Bonferonni method. In addition, receiver operating characteristic (ROC) curves were constructed to determine the optimal cut-off levels of the most significant parameters allowing differentiation between WT and PA. Myoepitheliomas and basal cell adenomas have been excluded from statistical analysis due to a small number of lesions. 

## 3. Results

ICCs of tumor derived features presented with excellent inter-rater reliability except for D*—good agreement; *p* value < 0.001—Table 3.

Values of magnetic resonance derived features of the most common benign parotid gland tumors and of contralateral healthy parotid parenchyma are gathered in Table 4. The ADC values of WTs were significantly lower than that of PAs. Pleomorphic adenomas showed significantly higher D and Ve values than Warthin tumors (*p* < 0.001). D* values and Kep values of Warthin tumors were significantly higher than that of PAs (*p* < 0.001). 

Kruskal–Wallis test for independent samples proved that statistically significant (*p* < 0.001) inter-group differences exist between PAs and WTs in all tested parameters but iAUC, pleomorphic adenomas differ from healthy parotid gland in all aspects while the values of D* and FP of Warthin tumors and healthy parotid glands are very similar—Table 5. The significance level was obtained at 0.05.

**Figure 1:**
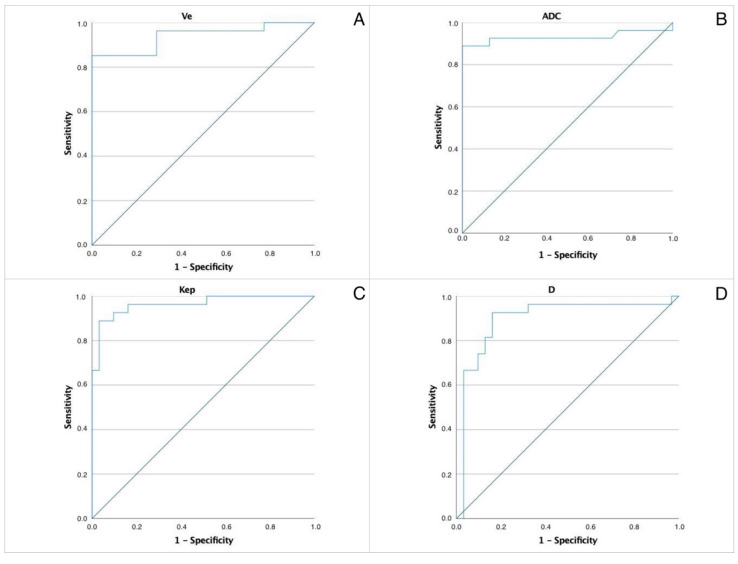
ROC of the most significant parameters allowing differentiation between WT and PA. AUC values and thresholds were for (**A**) ADC. AUC 0.931, threshold 1.05. (**B**) Pure diffusion coefficient (**D**) AUC 0.896, threshold 0.9. (**C**) Kep. AUC 0.964, threshold 1.1. (**D**) Ve. AUC 0.939, threshold 0.299.

ROC curves were constructed to determine the optimal cut-off levels of the most significant parameters allowing differentiation between WT and PA—Figure 1. The area under the curve (AUC) values and thresholds were for ADC: 0.931 and 1.05, D: 0.896 and 0.9, Kep: 0.964 and 1.1 and Ve: 0.939 and 0.299, respectively.

**Figure 2:**
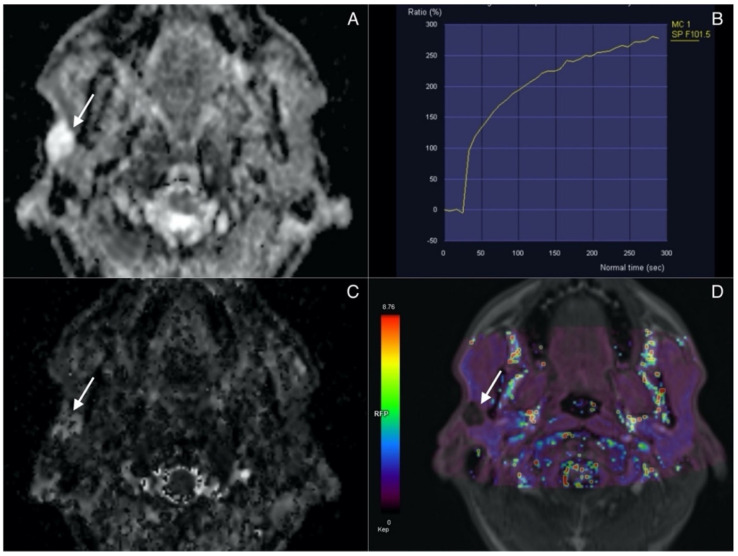
Pleomorphic adenoma (arrow) in superficial lobe of the right parotid. (**A**) ADC map. Lesion presents with high signal intensity correlating with lack of diffusion restriction. (**B**) Time Intensity Curve showing continuous contrast agent uptake consistant with type A curve and typical for adenomas. (**C**) IVIM. D map (high values). (**D**) Permeability. Kep map (low values).

Lesions presenting with combination of ADC, D, and Ve values superior to the cut-off and Kep values inferior to the cut-off are classified as pleomorphic adenomas. A representative case of PA is presented in Figure 2. 

**Figure 3:**
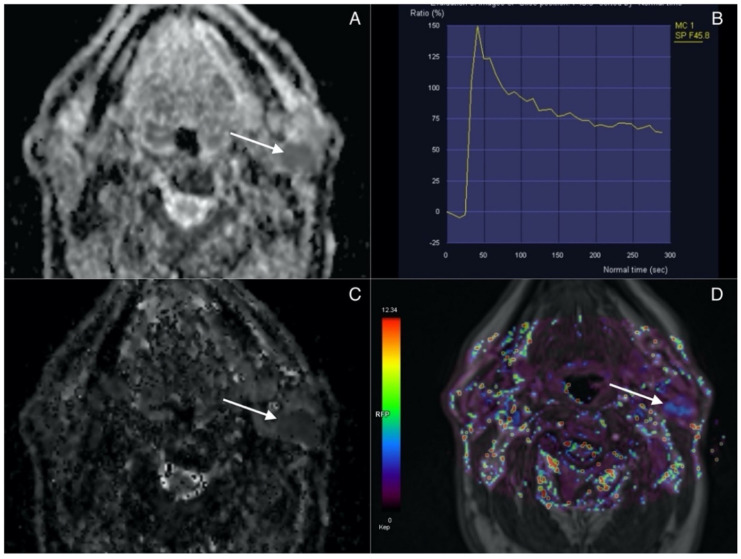
Warthin tumor (arrow) in superficial lobe of the left parotid. (**A**) ADC map. Lesion presents with low signal intensity correlating with diffusion restriction. (**B**) Time intensity curve showing rapid contrast agent uptake with high-rate washout corresponding to type B curve typical for WT. (**C**) IVIM. D map (low values). (**D**) Permeability. Kep map (high values).

Lesions presenting with combination of ADC, D, Ve values inferior to the cut-off and Kep values superior to the cut-off are classified as Warthin tumors. Figure 3 highlights the imaging features of WTs.

AUC for the rest of analyzed features presented as follows: Ktrans 0.692, D* 0.763, FP 0.773 and iAUC 0.576. The correlation between the area under the ROC curve and diagnostic accuracy is interpreted as 0.9–1.0 excellent, 0.8–0.9 very good, 0.7–0.8 good, 0.6–0.7 sufficient, 0.5–0.6 bad, respectively; with a value less than 0.5 representing that the test is not useful. Due to bad diagnostic accuracy value of AUC for the iAUC further analysis was not performed for that selected parameter.

Lesions presenting with combination of ADC, D, and Ve values superior to cut-off and Kep values inferior to cut-off are classified as pleomorphic adenomas. 

Lesions presenting with combination of ADC, D, and Ve values inferior to cut-off and Kep values superior to cut-off are classified as Warthin tumors. 

Diagnostic accuracy of IVIM- and DCE-derived parameters are shown in Table 6. 

When a combination of parameters (ADC, Kep and D) underwent analysis, we obtained diagnostic accuracy of 94.8% (95%CI 85.59–98.91), sensitivity of 92.59% (95%CI 75.71–99.09), specificity of 96.77% (95%CI 83.30–99,92), PPV 96.22% (95%CI 78.49–98.25) and NPV of 93.64% (95%CI 79.49–98.25). 

Diagnostic accuracy increased to 96.55% (95%CI 88.09–99.58) when we analyzed a combination of ADC, Kep and D*; sensitivity—96.30% (95%CI 81.03–99.91), specificity—96.77% (95%CI 83.30–99.92), PPV 96.36% (95%CI 79.36–99.45) and NPV of 96.72% (95%CI 81.13–99.51). 

However, we did not observe a further increase in diagnostic accuracy after including the fourth parameter (Ve) in the analysis. Accuracy in fact dropped to 91.33% (95%CI 80.95–97.11%) with sensitivity of 85.19% (95%CI 66.27–95.81), specificity of 96.77% (95%CI 83.30–99.92), PPV 95.90% (95%CI 77.19–99.39) and NPV of 88.05% (95%CI 74.84–94.80).

## 4. Discussion

Characterization of parotid focal lesions is crucial, as treatment differs accordingly to histopathological type of the tumor. Clinical findings provide limited information and FNAC is not always conclusive. Thus, pre-operative magnetic resonance imaging gains recognition in a pre-treatment diagnostic algorithm. Interpreted in conjunction with conventional morphological sequences, dynamic contrast-enhanced MRI and diffusion-weighted MRI have high potential for the characterization of parotid tumors [37], according to research comparable to that of FNAC [7,38]. Nowadays, novel advanced DWI analysis techniques are under ongoing research with the aim to identify characteristic features of different histopathological types of parotid tumors in order to further increase diagnostic accuracy [39]. In our study DWI, IVIM and quantitative analysis of DCE-MRI derived parameters demonstrated distinctive features of pleomorphic adenomas and Warthin tumors as confirmed with a Kruskal–Wallis test for independent samples. The test proved that statistically significant (*p* < 0.001) inter-group differences exist between pleomorphic adenomas and Warthin tumors in all tested parameters but iAUC. 

Pleomorphic adenomas presented with mean ADC value of 1.32 × 10^−3^ mm^2^/s which is in concordance to previous studies reporting a cut-off ADC value between 1.267 × 10^−3^ mm^2^/s and 1.60 × 10^−3^ mm^2^/s [40,41]. The ADC values of Warthin tumors were significantly lower than that of PAs. In our study the ADC cut-off value distinguishing between WTs and PAs was established at the level of 1.05 × 10^−3^ mm^2^/s (AUC 0.931) with diagnostic accuracy of 94.8%, sensitivity 88.9%, specificity 100%, PPV 100% and NPV 91%. A similar ADC value was obtained by Nada et al. with a cut-off 1.16 ± 0.31 (SD) × 10^−3^ mm^2^/s [42]. 

IVIM, allowing separate evaluation of perfusion and diffusion-related features of salivary gland tumors, may help discriminate parotid gland tumors with good and excellent accuracy. Warthin tumors presented with significantly lower D values than pleomorphic adenomas with a threshold value equal or less than 0.9 × 10^−3^ mm^2^/s. Previously, Sumi et al. [43] in their prospective study including 12 pleomorphic adenomas and 8 Warthin tumors evaluated the D cut-off value at the level of 1.1 × 10^−3^ mm^2^/s allowing differentiation of WT from PAs with 100% accuracy, specificity and sensitivity. As the authors imply these results may be attributed to large areas of densely packed small lymphocytes in Warthin tumors, which greatly limit pure diffusion. D* values of WTs were significantly higher than in case of PAs. D* reflects tumor vascularity being proportional to the average blood velocity and mean capillary segment length [8]. 

The role of quantitative parameters of DCE-MRI reflecting the microvascular density of the lesion is less studied than IVIM. Warthin tumors showed significantly higher Kep values than pleomorphic adenomas (the median value was 2.49 min^−1^). This is in concordance with results obtained by Yabuuchi et al. [30] In their study on the added value of permeability imaging Kep, representing a rate constant between the extravascular space and the blood plasma, proved to be the only significant feature derived from DCE-MRI. Further the authors interpolated the results on TIC pattern, speculating that high Kep values correspond to a high wash-out rate, typically seen in case of WTs [30]. Additionally, in our study 93.5% (29 tumors out of 31 total WTs) of Warthin tumors showed a type B time intensity curve, characterized by an early enhancement (T_peak_ prior to 120 s) and high washout pattern (WR over 30% at 5 min). In addition, the Ve values of WTs was significantly lower that than of pleomorphic adenomas. Taking that into consideration, we agree with Yabuuchi et al. that further analysis of Kep and Ve parameters may prove them feasible for precise evaluation of the contrast agent movement between extravascular extracellular space and blood pool [28]. Furthermore, in the light of recent research by Xu et al., Ve seems to have a discriminative potential between benign and malignant lesions [39].

The ROC analysis allowed us to propose the following thresholds allowing to differentiate WTs from PAs: ADC 1.05 (AUC 0.931), D 0.9 (AUC 0.896), Kep 1.1 (AUC 0.964) and Ve 0.299 (AUC 0.939). Lesions presenting with combination of ADC, D, and Ve values superior to the cut-off and Kep values inferior to the cut-off are classified as pleomorphic adenomas. Lesions presenting with combination of ADC, D, and Ve values inferior to the cut-off and Kep values superior to the cut-off are classified as Warthin tumors. 

Similarly to Patella et al. [33], we found that a combination of analyzed parameters increases diagnostic accuracy and its indices, however the optimal combination included no more than three features (with the best values obtained with ADC, Kep and D or ADC, Kep and D*).

The authors are aware of the following limitations. Firstly, it was a single-center study. Secondly, the sample size calculation has not been conducted as we recruited all consecutive patients within certain time period about inclusion and exclusion criteria. Additionally, due to a small number of lesions as well as group heterogeneity malignant lesions needed to be excluded from the study. However, we are now collecting cases for a validation study and researching parotid malignancies. At this point we have not yet analyzed different histopathological subgroups of PAs and WTs; however, as this study is a part of an ongoing prospective research, we will hopefully be able to investigate if and at to what extend the analyzed features may be dependent on pathological subtype of the tumor. 

## 5. Conclusions

In our study IVIM and quantitative DCE-MRI derived parameters demonstrated distinctive features of PAs and WTs and as such they seem feasible in differentiation of benign parotid gland tumors in a multiparametric magnetic resonance approach. With a precise pre-treatment diagnostic tool, patients can be offered optimal treatment.

## Figures and Tables

**Table 1:** Final study cohort demographic data.

		Warthin Tumors	Pleomorphic Adenomas	Myoepitheliomas	Basal Cell Adenomas
Sex	Females	17	19	3	2
	Males	14	8	0	1
Total number of lesions		31	27	3	3
Mean BMI	Females	29.2	26.3	25.3	24.9
	Males	32.4	23.7	-	26.5
Side	Right	15	18	-	1
	Left	16	9	3	2
Location	Superficial lobe	25	15	-	2
	Deep lobe	6	10	3	-
	Accessory lobe	-	2	-	1
Size * (mm)	Females	17	17	38	8
	Males	20	15	-	19
Lymphadenopathy	Females	6	1	1	1
	Males	7	2	-	1
Facial nerve palsy	Females	0	0	0	0
	Males	0	1 (post-operative)	0	0

* Mean biggest diameter in axial plane.

**Table 2:** Imaging protocol.

Sequence	Orientation	TR (ms)	TE (ms)	SL/SPC	FOV	Matrix
T2 Blade	Sagittal	4070	144	3.0/3.6	250∗250	320∗320
T2 TIRM	Coronal	6000	100	3.0/3.6	229∗271	540∗640
T1 TSE	Transverse	555	11	3.0/3.6	195∗240	416∗512
T2 TSE DIXON	Transverse	3050	75	3.5/4.0	218∗240	442∗608
ep2d_DWI	Transverse	8130	65	4.0	236∗250	208∗220
ep2d_DWI ADC	Transverse	8130	65	4.0	236∗250	208∗220
T1 Vibe DIXON	Transverse	7.7	2.4	1.0i	189∗220	163∗224
T1 Vibe CM Dyn 1–36	Transverse	4.2	1.6	3.5i	260∗260	154∗192
T1 Vibe DIXON CM	Transverse	7.7	2.4	1.0i	189∗220	163∗224

TIRM: turbo inversion recovery magnitude; Vibe: volumetric interpolated breath-hold examination; TSE: Turbo Spin Echo; DWI: diffusion-weighted imaging; ADC: apparent diffusion coefficient; CM: contrast medium; TR: repetition time; TE: echo time; SL: slice thickness; FOV: field of view; i: isotropic.

**Table 3:** Results of the ICC analysis.

	IVIM				Permeability				
	D	D*	FP	ROI	Ktrans	Kep	Ve	iAUC	ROI
ICC	0.963	0.827	0.925	0.997	0.963	0.990	0.940	0.967	0.995
95% CI	0.957–0.984	0.715–0.895	0.876–0.955	0.995–0.998	0.940–0.978	0.984–0.994	0.901–0.963	0.946–0.980	0.991–0.998

*p* value < 0.001. ICC: intra-class correlation coefficient; CI: Confidence Interval; D: pure diffusion coefficient; D*: pseudo-diffusion coefficient; FP: fraction perfusion; Ktrans: transfer constant; Kep: reflux constant; Ve: extra-vascular extra-cellular volume fraction; iAUC: initial area under curve in 60 s.

**Table 4:** Magnetic resonance features of common benign parotid gland tumors.

		Warthin Tumors		Pleomorphic Adenomas		Contralateral Parotid	
Magnetic Resonance Parameters		Median	Interquartile Range	Median	Interquartile Range	Median	Interquartile Range
ADC 10^−3^ mm^2^/s		0.68	0.1	1.32	0.25	0.84	0.21
IVIM	D 10^−3^ mm^2^/s	0.63	0.17	1.19	0.53	0.71	0.12
	D*10^−3^ mm^2^/s	135.9	145.5	47.05	20.7	117.76	41.89
	FP %	24.8	8.5	34.4	14.5	23.6	8.3
Permeability	Ktrans min^−1^	0.41	0.29	0.17	0.34	0.13	0.14
	Kep min^−1^	2.49	1.04	0.31	0.81	1.22	0.49
	Ve	0.16	0.1	0.62	0.76	0.12	0.09
	iAUC	0.27	0.11	0.24	0.38	0.14	0.15
		No of lesions		No of lesions			
TIC	Type A	0		19			
	Type B	29		1			
	Type C	2		7			

ADC: Apparent Diffusion Coefficient; IVIM: Incoherent Voxel Motion; D: pure diffusion coefficient; D*: pseudo-diffusion coefficient; FP: fraction perfusion; Ktrans: transfer constant; Kep: reflux constant; Ve: extra-vascular extra-cellular volume fraction; iAUC: initial area under curve in 60 s; TIC: Time Intensity Curve.

**Table 5:** Kruskal–Wallis test for independent samples.

Sample 1/Sample 2		Pleomorphic Adenomas vs. Warthin Tumors	Pleomorphic Adenomas vs. Healthy Parotids	Warthin Tumors vs. Healthy Parotids
ADC		<0.001	<0.001	<0.001
IVIM	D	<0.001	<0.001	0.049
	D*	<0.001	<0.001	0.922
	FP	<0.001	<0.001	0.52
Permeability	Ktrans	0.004	0.042	<0.001
	Kep	<0.001	<0.001	<0.001
	Ve	<0.001	<0.001	0.004
	iAUC	0.319	<0.001	<0.001

ADC: apparent diffusion coefficient; IVIM: incoherent voxel motion; D: pure diffusion coefficient; D*: pseudo-diffusion coefficient; FP: fraction perfusion; Ktrans: transfer constant; Kep: reflux constant; Ve: extra-vascular extra-cellular volume fraction; iAUC: initial area under curve in 60 s.

**Table 6:** Diagnostic accuracy of IVIM- and DCE-derived parameters.

		Sensitivity %	95% CI	Specificity %	95% CI	PPV %	95% CI	NPV %	95% CI	Accuracy %	95% CI
ADC		88.9	79.8–97.7	100	88.8–100	100	NA	91.0	77.7–96.7	94.8	85.6–98.9
IVIM	D	81.5	61.9–93.7	87.1	70.2–96.4	84.9	68.8–93.4	84.1	70.4–92.2	84.5	72.6–92.6
	D*	85.2	66.3–95.8	70.9	52.0–85.8	72.24	59.5–82.2	84.4	68.0–93.2	77.7	64.8–87.5
	FP	48.2	28.7–68.1	93.6	78.6–99.2	86.9	62.1–96.4	67.1	58.3–74.8	72.2	58.9–83.2
Permeability	Ktrans	44.4	25.5–64.7	100	88.8–100	100	NA	66.9	59.2–73.9	73.9	60.7–84.5
	Kep	88.9	70.8–97.7	96.8	83.3–99.9	96.1	77.9–99.4	90.1	77.1–96.6	93.1	83.2–98.1
	Ve	85.2	66.3–95.8	100	88.8–100	100	NA	88.4	75.5–94.9	93.1	83.2–98.1
	iAUC	NA	NA	NA	NA	NA	NA	NA	NA	NA	NA

ADC: apparent diffusion coefficient; IVIM: incoherent voxel motion; CI: confidence interval; D: pure diffusion coefficient; D*: pseudo-diffusion coefficient; FP: fraction perfusion; Ktrans: transfer constant; Kep: reflux constant; Ve: extra-vascular extra-cellular volume fraction; iAUC: initial area under curve in 60 s.

## Data Availability

Data available on request due to restrictions (privacy). The data presented in this study are available on request from the corresponding author. The data are not publicly available due to above mentioned restrictions.
